# Single-Event Transients in an IEEE 802.15.4 RF Receiver for Wireless Sensor Networks

**DOI:** 10.3390/s20164399

**Published:** 2020-08-06

**Authors:** Sergio Mateos-Angulo, Javier del Pino, Daniel Mayor-Duarte, Mario San-Miguel-Montesdeoca, Sunil L. Khemchandani

**Affiliations:** 1 Wireless Innovative MMIC (WIMMIC) S.L., 35004 Las Palmas de Gran Canaria, Spain; smateos@iuma.ulpgc.es (S.M.-A.); daniel.mayor@wimmic.com (D.M.-D.); mario.sanmiguel@wimmic.com (M.S.-M.-M.); 2 Institute for Applied Microelectronics (IUMA), University of Las Palmas de Gran Canaria, 35017 Las Palmas de Gran Canaria, Spain; sunil.lalchand@ulpgc.es

**Keywords:** CMOS, filter, mixer, low-noise amplifier, radiation, RF receiver, Single Event Transients, Wireless Sensor Networks

## Abstract

This paper presents a procedure to analyse the effects of radiation in an IEEE 802.15.4 RF receiver for wireless sensor networks (WSNs). Specifically, single-event transients (SETs) represent one of the greatest threats to the adequate performance of electronic communication devices in high-radiation environments. The proposed procedure consists in injecting current pulses in sensitive nodes of the receiver and analysing how they propagate through the different circuits that form the receiver. In order to perform this analysis, a Complementary Metal Oxide Semiconductor (CMOS) low-IF receiver has been designed using a 0.18 μm technology from the foundry UMC. In order to analyse the effect of single-event transients in this receiver, it has been studied how current pulses generated in the low-noise amplifier propagate down the receiver chain. The effect of the different circuits that form the receiver on this kind of pulse has been studied prior to the analysis of the complete receiver. First, the effect of SETs in low-noise amplifiers was analysed. Then, the propagation of pulses through mixers was studied. The effect of filters in the analysed current pulses has also been studied. Regarding the analysis of the designed RF receiver, an amplitude and phase shift was observed under the presence of SETs.

## 1. Introduction

Wireless sensor networks (WSNs) have allowed the implementation of interconnected electronic systems in a wide number of fields such as industry, environmental applications or medicine [1,2,3,4,5,6,7,8,9,10]. However, there still exist several applications in which high radiation represents an obstacle for the use of this kind of network [11]. Specifically, sectors such as aeronautics, aerospace, nuclear and health will benefit from its use. As an example, the use of WSNs will reduce the wiring for intra-satellite communications in aerospace applications, thus reducing the weight and volume. This is a key aspect for satellites, as it reduces the cost of launching them into space. Additionally, the flexibility of the communication networks is enhanced as less time is required for assembly, integration and testing of the sensor nodes.

In high-radiation environments, high-energy ionising particles can produce adverse effects in electronic devices. The two main effects that can be produced are total ionising dose (TID) and single-event effects (SEEs). The first gradually degrades the performance of the device due to the accumulation of generated charges. Regarding SEEs, they produce current and voltage peaks when a high-energy particle strikes a semiconductor device [11]. In order to mitigate the damages produced by these radiation effects in WSNs, metal shielding can be utilised. However, this shielding must be lightweight so that the total load of the spacecraft is not considerably increased. Taking this into account, the shielding cannot fully stop the high-energy particles from reaching the electronics. Therefore, it is a major goal in the radiation effects research community to develop radiation-hardened sensor devices, robust circuits and systems that can operate in high-radiation environments [12,13,14,15,16]. Radiation hardening techniques can be implemented at different levels. Radiation-hardening-by-design (RHBD) is the most extended solution as it meets specified radiation performance criteria without modifying the existing technology process and maintaining the device electrical behaviour [17].

Considering this, radiation tolerance in electronics has become a relevant issue in the field of communication systems, specially for electronics in harsh environments. Many solutions for radiation-tolerant circuits can be found implemented in gallium-nitride (GaN), silicon-germanium (SiGe) or silicon-on-insulator (SOI) processes [18,19,20,21,22,23,24,25]. Regarding GaN devices, their wide bandgap, large breakdown electric field and outstanding thermal stability make them an attractive candidate for space applications [20]. Several studies have concluded that GaN transistors have a high tolerance to accumulative dose effects [21,22]. Nevertheless, other studies have shown some vulnerability in GaN transistors against SEEs [19].

Silicon-germanium transistors have been receiving more attention in recent years, as higher integration levels, higher yield and lower costs can be obtained compared to GaN-based transistors [11]. Furthermore, SiGe transistors possess a higher tolerance to TID when compared to Complementary Metal Oxide Semiconductor (CMOS) devices, where the dielectric oxide layers are considerably more vulnerable to damages produced by radiation exposure over long periods of time. However, SiGe devices are vulnerable against SEEs [24]. Taking this into account, several studies regarding SEEs in SiGe devices have proliferated in recent years [24,25,26].

Regarding SOI processes, it has been found that they have a better response to single-event effects compared to bulk CMOS processes [23]. This can be mainly explained due to less depletion region and the existence of the buried oxide, which reduces the silicon volume where incident radiation results in charge generation [27].

Despite the advantages of using GaN, SiGe or SOI processes, CMOS technologies provide a much more cost-efficient solution [28]. These technologies are not inherently tolerant to radiation, but they can be suitable for harsh environment applications by implementing RHBD techniques [29]. Additionally, CMOS processes have become more robust against TID due to device scaling. In advanced CMOS technologies, the gate oxide thickness has decreased considerably, which leads to less charges being trapped in the gate oxide of the transistors [17]. However, SEEs have become more problematic in CMOS technologies due to device scaling, as particles with less energy are able of producing SEEs [29]. Taking this into account, this work focuses on the analysis of SEEs in an RF CMOS receiver. Specifically, single-event transients (SETs) are analysed as they are particularly troublesome in analogue circuits.

Even though SETs have mainly been studied in digital circuits, they represent one of the greatest liabilities in high-frequency analogue circuits. Additionally, wireless communication systems have not been thoroughly studied under these effects due to the difficulty in measuring and evaluating their influence on system performance. The authors of [30] propose an approach where the SET performance of an RF SiGe receiver is characterised by using a periodic SET pulsed source to evaluate the distortion of the simulated constellation (I-Q) diagram. However, this implies that there will be a SET strike at every symbol period. This is generally not the case, which leads to the necessity of using some other kind of study. For example, the symbol rate specified in the IEEE 802.15.4 standard is 62.5 KHz, which means that there is a symbol every 16 μs. Taking into account that the pulses analysed in this work last only a few nanoseconds, the effect of the pulse will have disappeared when the symbol is sampled, unless the SET occurs at the exact same time at which the symbol is being sampled. Therefore, in this paper a procedure is proposed where the voltage signal is analysed at the critical nodes of the receiver chain, searching for possible variations in the signal.

**Figure 1:**
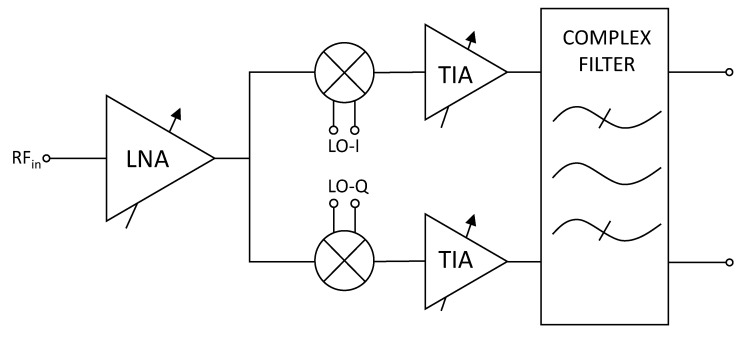
Architecture of a typical low-IF receiver.

Specifically, a CMOS low-IF receiver designed using a UMC 0.18 μm technology is presented in this paper. However, before analysing the designed receiver, the conventional architecture of a low-IF receiver is addressed. Figure 1 shows the typical architecture of a low-IF receiver. As it can be seen, it is composed of a low-noise amplifier (LNA), which is followed by a down-conversion mixer. The output current of the mixer is converted to voltage by the transimpedance amplifier (TIA) and is finally filtered by a complex filter.

In order to fully analyse how a pulse propagates through a receiver, the effect on SETs of the different circuits that compose the receiver has been studied. In this case, the effects generated in the LNA are studied as they propagate through the receiver chain. The methodology that has been employed consists of applying current pulses in the critical nodes of the LNA and analysing the voltage signal at different nodes of the receiver. Taking this into account, the effect of SETs on low-noise amplifiers is addressed in Section 2. A more extensive and thorough analysis regarding LNAs has been performed in some of our previous work [31]. In this paper, only the most important aspects of said paper are presented. The influence of LC tank circuits on SETs is studied in Section 3. Section 4 studies the effect of down-conversion mixers on SETs, while the SET performance of filters is investigated in Section 5. After the individual circuits have been studied, the analysis of the effect of SETs on the designed receiver is performed in Section 6. Finally, some conclusions are given in Section 7.

## 2. Single Event Transients in Low-Noise Amplifiers

**Figure 2:**
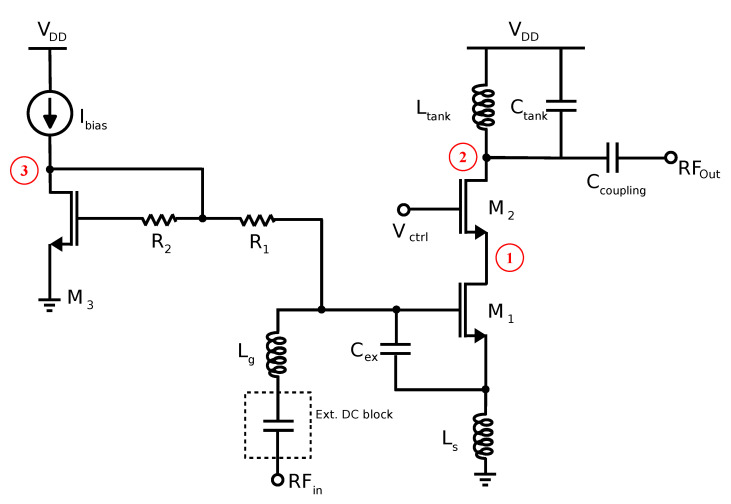
Schematic of the common-source cascode LNA.

In this section, an analysis of the effect of radiation in low-noise amplifiers is performed. As mentioned previously, a thorough analysis regarding LNAs was performed in [31]. In said paper, an analysis of different LNA topologies was carried out. Specifically, a common-gate and common-source LNA were studied under the effects of radiation. In this paper, only the LNA that follows a common-source with inductive degenerated cascode topology is studied, as the LNA included in the receiver presented in this work follows this structure. This topology is a well-known and widely used structure in RF receivers. Figure 2 shows the schematic of the designed LNA, which is also included in the designed receiver analysed in Section 6.

In order to analyse the effect of radiation in this LNA, the methodology presented in [31] is used. This methodology consists in employing a physics-based technology computer-aided design (TCAD) software tool to model semiconductor devices and perform ion strike simulations to study how they affect the device performance. The results that are obtained in these simulations are then used in an electrical circuit domain simulator. This way the accuracy of the device simulator is combined with the fast simulations performed in the circuit domain simulator. This methodology has already been proven in [25], where the simulation results closely match the obtained measurement results.

**Figure 3:**
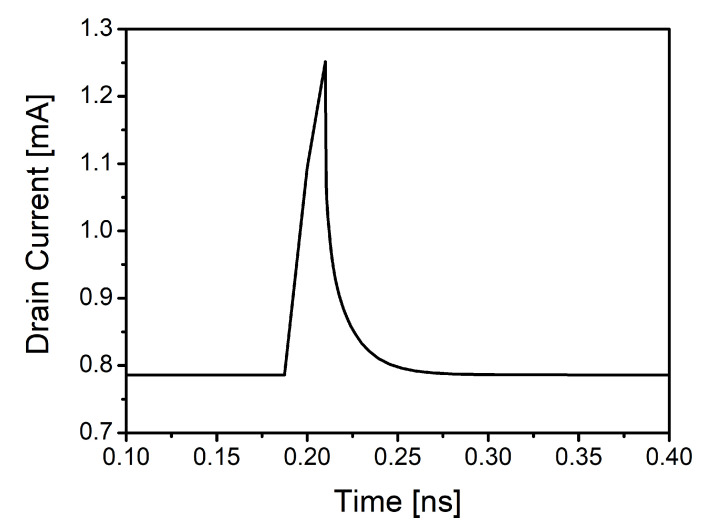
Technology computer-aided design (TCAD)-simulated current pulse in the drain of the transistor due to a heavy ion impact.

In this case, the physics-based TCAD simulator Sentaurus by Synopsys was used to model an NMOS transistor of the UMC 0.18 μm technology and to perform heavy ion simulations. The heavy ion model of this tool was used to simulate the effect of a charged particle striking the modelled transistor. This model is commonly used to perform this kind of SET simulations [23,25,32]. Furthermore, it is known that the most critical areas of an NMOS transistor when there is an ion impact are the reverse-biased junctions, which correspond to the *n-p* junction between the drain and substrate [17]. Figure 3 shows the TCAD-simulated current pulse generated at the drain of the modelled transistor due to the impact of a heavy ion for a specific linear energy transfer (LET) and penetration depth of the particle in the device [31]. As it can be seen, the generated pulse has approximately a double-exponential shape that can be modelled following (1),

(1) $$I_{rad}=\frac{Q}{t_{f}-t_{r}}\cdot \left(e^{-\frac{t}{t_{f}}}-e^{-\frac{t}{t_{r}}}\right)$$

where *Q* is the collected charge, and $t_{f}$ and $t_{r}$ are the fall and rise time, respectively. This is in concordance with a typical model used to simulate the effects of SETs in CMOS technologies, based on introducing double-exponential current pulses in critical nodes of the circuits [17,33].

The TCAD-generated current pulses were then introduced in the circuit domain simulator Advanced Design System (ADS) by Keysight to analyse the most critical nodes of the LNA. Specifically, the current pulses were applied at nodes 1, 2 and 3, as seen in Figure 2. The maximum voltage peak and the recovery time of the output signal were calculated for each case. The output signal is considered to be recovered when the difference in the output voltage signal in the case of a SET strike and the case of no strike is below 5% of the maximum output voltage peak. Taking this into account, simulation results show that the biasing network (node 3) is the most vulnerable node of the LNA [31]. This can be explained by the fact that any voltage variation induced by an ion strike in the bias circuit will result in a deviation of the operating point of the transistors [17]. Additionally, the largest voltage peaks are obtained when a strike occurs at node 2. This happens because this node is directly connected to the output of the LNA.

## 3. LC Tank Circuits Influence on SETs

**Figure 4:**
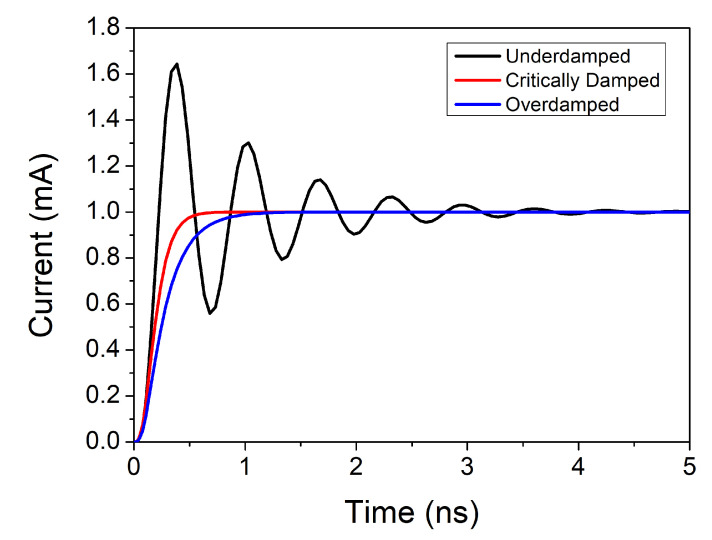
Step response of an resistor-inductor-capacitor (RLC) parallel circuit.

In this section, the influence of an LC tank circuit on SETs generated in the LNA is analysed. In essence, an LC tank circuit is a resistor-inductor-capacitor (RLC) parallel circuit. Therefore, in order to perform this analysis, the step response of an RLC parallel circuit is studied. Figure 4 shows the step response of an RLC parallel circuit. As stated in basic circuit theory, the step response depends on the *α* and *ω*_0_ parameters:

(2) $$\alpha =\frac{1}{2RC}$$

(3) $$\omega_{0}=\frac{1}{\sqrt{LC}}$$

Depending on the values of *α* and *ω*_0_, the response of a parallel RLC circuit can be classified into overdamped, critically damped or underdamped:

- *α* > *ω*_0_: Overdamped
- *α* = *ω*_0_: Critically damped
- *α* < *ω*_0_: Underdamped

However, the quality factor (Q) of an LC tank is typically approximately 10. Substituting in (4) and then in (2) and (3), we obtain *α* smaller than *ω*_0_. Under these conditions, the step response would be an underdamped response. This will be studied in greater detail in Section 6.

(4) $$Q=R\sqrt{\frac{C}{L}}$$

## 4. Mixers Influence on SETs

**Figure 5:**
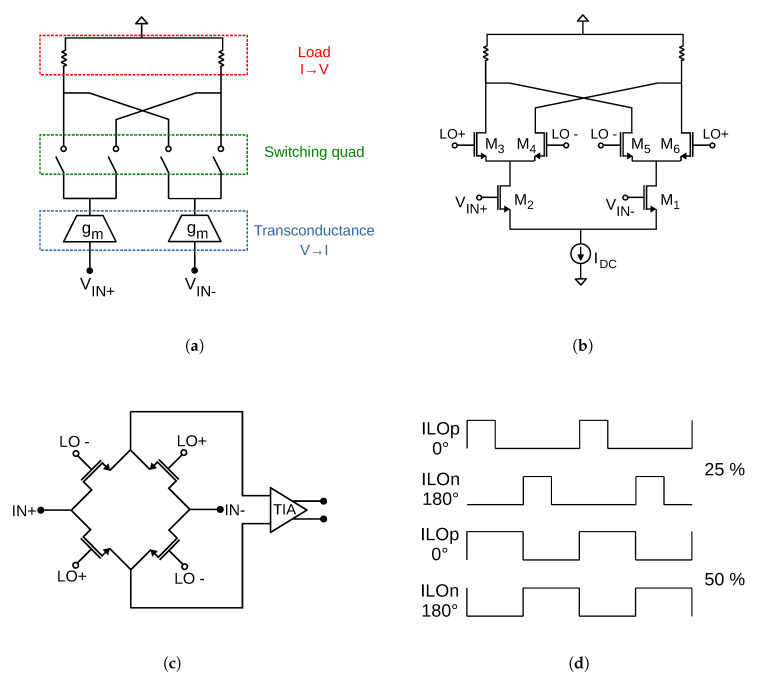
Schematic of a (**a**) ideal mixer, (**b**) Gilbert cell and (**c**) double-balanced passive mixer plus TIA. (**d**) Quadrature LO waveforms with 25% vs. 50% duty cycle.

In this section, the influence of mixers on SETs generated in the LNA is analysed. Figure 5a shows the diagram of an ideal mixer. As it can be seen, the mixer is formed by three main stages: transconductance, switching quad and load. The first is in charge of the voltage to current conversion at the input of the circuit. The current signal is then mixed in the switching quad which is driven by a local oscillator (LO) signal. This produces an output signal with a frequency equal to the difference between the RF input frequency and the LO frequency, in the case of down-conversion mixers. This output signal is then converted to voltage in the load of the mixer.

A typical implementation of this ideal structure is the Gilbert cell, which is shown in Figure 5b. The transconductance stage is formed by transistors M_1_ and M_2_ acting as transconductors, while transistors M_3–6_ commutate the RF input to the outputs. The load can be implemented in several ways (resistive load, active load, etc.). In this case, the load is depicted as a resistive load for simplicity.

Another implementation of the ideal mixer is shown in Figure 5c. In this case, a double-balanced passive mixer is shown, where the load is implemented as a transimpedance amplifier (TIA). The main aim of this amplifier is to convert current into voltage, which must be achieved in the load of the mixer.

Taking this into account, a theoretical study of how SETs generated at the LNA propagate through an ideal mixer that follows the structure shown in Figure 5a has been performed. The results obtained in this study can be transferred to any other mixer implementation, such as those exposed in Figure 5b,c. For the sake of simplicity, the current pulses are considered to have an ideal rectangular shape instead of the double-exponential shape studied in the LNA. The conclusions obtained in this study can be considered to remain true for other pulse shapes [32]. Additionally, an ideal Butterworth low-pass filter, with a passband edge frequency of 1 GHz and a stopband edge frequency of 1.2 GHz, has been included at the output of the mixer to select the down-converted signal.

In order to understand how this pulse will propagate to the output of the circuit, it should be noted that the switches are changing from the ON-state to the OFF-state rapidly with the frequency of the local oscillator (LO) signal. In this case, the LO frequency is 2 GHz. At a particular instant, two of the switches are in the ON-state and the other two are in the OFF-state. Consequently, the transient current finds a path to the outputs of the mixer. At another time instant, the opposite happens (the switches that were in the ON-state are now in the OFF-state, and vice versa). This occurs periodically, with the period of the LO signal. Taking this into account, it can be considered that the switches are sampling the transient current at each instant [32]. Therefore, the pulse will propagate to the output of the circuit.

As stated in [32], for the original shape of the pulse to propagate to the output of the circuit, the following condition must be met,

(5) $$f_{LO}>\frac{2}{\tau }$$

where τ is the width of the current pulse and *f_LO_* is the frequency of the local oscillator.

**Figure 6:**
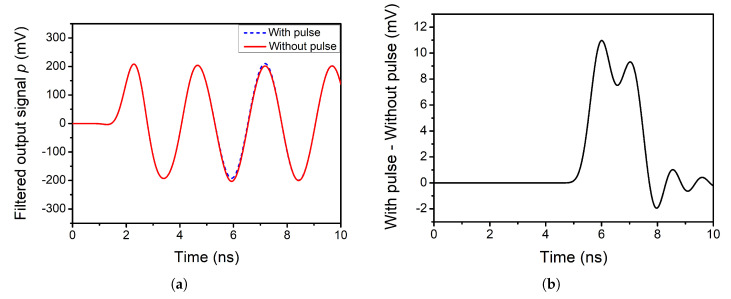
(**a**) Filtered single-ended output of the double-balanced mixer when there is a pulse at the input (τ=2 ns) and when there is no pulse. (**b**) Effect of the pulse on filtered single-ended output (τ=2 ns).

Considering that in this case the *f_LO_* is 2 GHz, the pulse width is set to τ=2 ns. The output signals of the mixer are analysed under these conditions. Figure 6a shows the output of the positive branch after the filtering stage. The dashed blue line represents the case when a pulse is introduced and the solid red line the case when there is no pulse. Regarding Figure 6b, it shows the difference between these two cases for the positive branch.

As it can be seen, the obtained pulse resembles the original pulse introduced at the input of the circuit. It must be noted that the exact shape of the ideal pulse is not seen, as part of the information is discarded during the filtering process. The same occurs for the negative branch.

### Effect of Duty Cycle

Until now, the duty cycle of the LO signal was set to the conventional value of 50%. However, it is known that employing a 25% duty cycle enhances the gain of a mixer by approximately 3 dB [34]. In this subsection, the SET performance of the double-balanced mixer is analysed when the duty cycle of the LO signal is set to 25%. Figure 5d shows the waveforms for a 25% and a 50% duty cycle.

**Figure 7:**
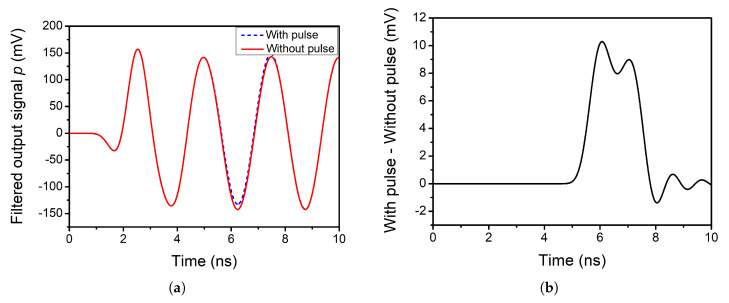
(**a**) Filtered single-ended positive output of the double-balanced mixer with 25% duty cycle when there is a pulse at the input (τ=2 ns) and when there is no pulse. (**b**) Effect of the pulse on filtered single-ended positive output of the mixer with 25% duty cycle when there is a pulse at the input (τ=2 ns).

Regarding the propagation of the pulse through the filtering stage, Figure 7a,b shows the obtained results for the positive branch. It can be seen that the double-balanced mixer with a 25% duty cycle behaves similarly in terms of SET performance as for the case of 50% duty cycle. Therefore, it can be stated that using a 25% duty cycle enhances the gain of the mixer without worsening its SET performance.

## 5. Filters Influence on SETs

In this section, the pulses that have been generated in the LNA and propagated through the mixer are analysed when they pass through a filter. As it was previously mentioned, filtering stages affect the SET performance as part of the information of the current pulses is discarded in the filtering process. In this section, the equations behind this concept are analysed. To do so, an ideal rectangular pulse is considered.

This ideal square pulse can be defined as [35]

(6) $$x\left(t\right)=\left\{\begin{array}{c} 1,\left|t\right|<\frac{\tau }{2}\\ 0,\left|t\right|>\frac{\tau }{2} \end{array}\right..$$

Applying the Fourier Transform

(7) $$X\left(j\omega \right)=\int_{-\infty }^{\infty }x\left(t\right)e^{-j\omega t}dt,$$

the following frequency response is obtained,

(8) $$X\left(j\omega \right)=\int_{-\frac{\tau }{2}}^{\frac{\tau }{2}}e^{-j\omega t}dt=2\frac{\sin\frac{\omega \tau }{2}}{\omega }.$$

This response can be expressed in terms of the *sinc* function:

(9) $$X\left(j\omega \right)=\frac{2\tau }{2}sinc\left(\frac{\frac{\omega \tau }{2}}{\pi }\right).$$

**Figure 8:**
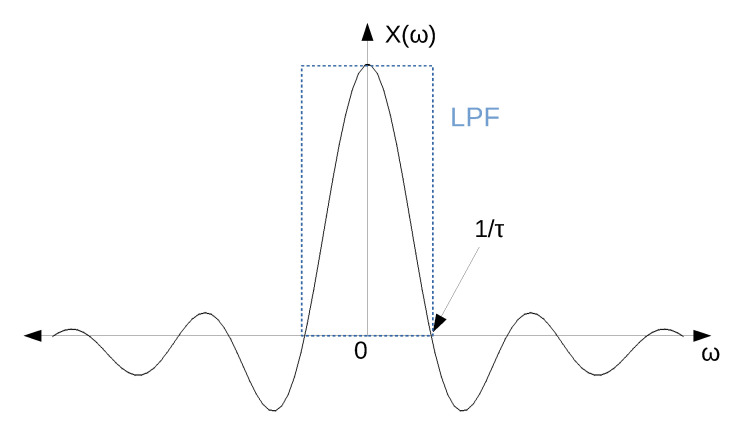
Frequency response of a square pulse and an ideal low-pass filter.

Figure 8 shows a sketch of the frequency response of the ideal pulse (X(jω)). As it can be seen, this function has a crossing by 0 at 1/τ and is centred at 0 Hz.

If only positive frequencies are considered, it can be stated that most of the information of the pulse can be found between 0 and 1/τ. This corresponds with the main lobe of the *sinc* function. Taking this into account, the filter must be able to capture the main lobe in order for the pulse to propagate to the output of the filter. This is represented in Figure 8, where an ideal low-pass filter (LPF) with a cut-off frequency at 1/τ is depicted.

In this particular case, a pulse with almost the same shape as the original pulse can be obtained at the output of the filter. In order to obtain the exact same shape, the cut-off frequency of the filter should be greater to capture more lobes of the *sinc* function. In any case, in this study it is considered that the pulse propagates to the output when the main lobe is captured, and therefore the shape of the output pulse resembles the square pulse.

However, if a band-pass filter (BPF) is used instead of a LPF, the information captured by the filter is different. The amount of information captured by the BPF will depend on its centre frequency and its bandwidth. In this work, narrow-band receivers have been considered. Specifically, receivers for low-power consumption wireless sensor networks have been studied (i.e., receivers for Bluetooth, ZigBee, etc.). In these standards, the bandwidth has a value of a few MHz. Therefore, for a square pulse with a width of a few nanoseconds, the BPF will only capture part of the main lobe, which results in a loss of spectral information and a minimised pulse at the output.

## 6. Single Event Transients in a Conventional Low-IF Receiver

In this paper, a procedure to analyse how a conventional low-IF RF receiver behaves under the effects of radiation is presented. The main focus of this procedure is to understand how a current pulse propagates through the different circuits that form the receiver. To do so, the voltage signal is analysed at the critical nodes of the receiver chain, searching for possible variations in the signal.

**Figure 9:**
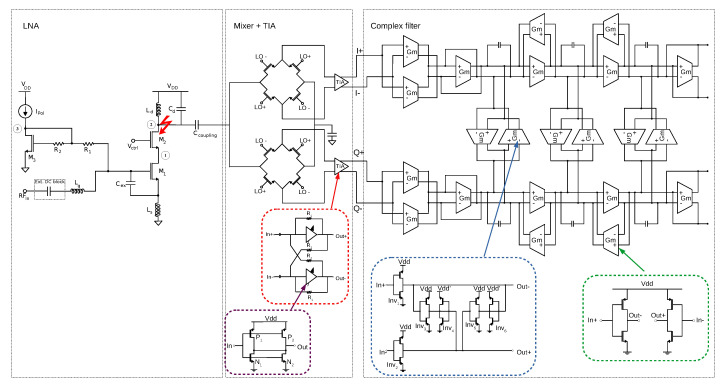
Architecture of the proposed low-IF conventional receiver.

**Figure 10:**
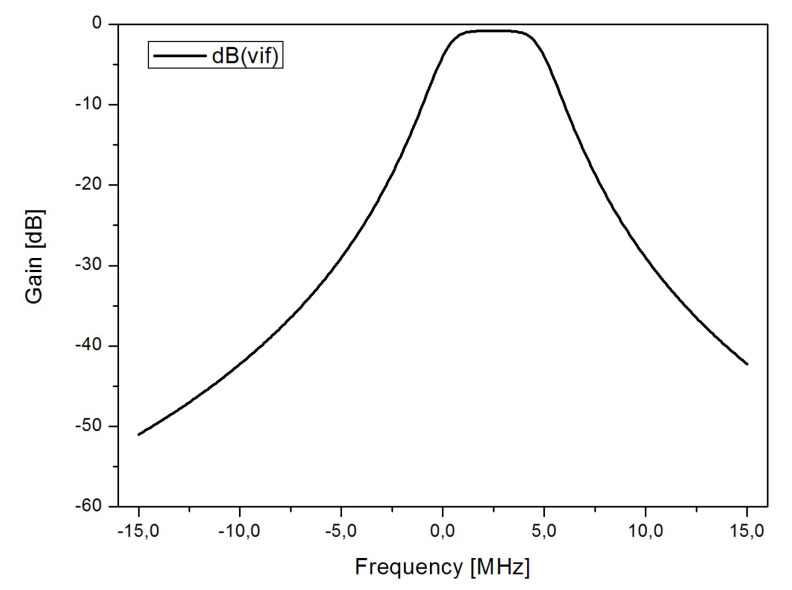
Frequency response of the complex filter.

The designed conventional low-IF architecture presented in [36] is studied. Figure 9 shows the schematic of the receiver architecture. The first element of the receiver is a low-noise amplifier, which is followed by a down-conversion mixer. The output current of the mixer is converted to voltage by the transimpedance amplifier (TIA) and is finally filtered by a complex filter. In this case, a Butterworth third-order gm-C complex bandpass filter has been designed. The structure is composed of two Butterworth third-order gm-C low-pass filters for the I and Q paths and two crossing extra signal paths per integrator to transform the low-pass prototypes to their bandpass complex counterparts. Inverter-based transconductors have been used in the I and Q paths, while Nauta’s transconductors have been implemented for the crossing signal paths that connect the I and Q branches. The frequency response of the filter is shown in Figure 10.

In this study, an analysis of how current pulses generated in the LNA propagate through the receiver is carried out. To do so, the double exponential pulses generated with the Sentaurus tool, which have been presented in Section 2, are employed. Specifically, the pulse with the highest maximum current peak is introduced at the drain of the output transistor of the LNA, where the largest voltage peaks at the output were obtained. The behaviour of the voltage signal is studied as it travels down the receiver chain.

**Figure 11:**
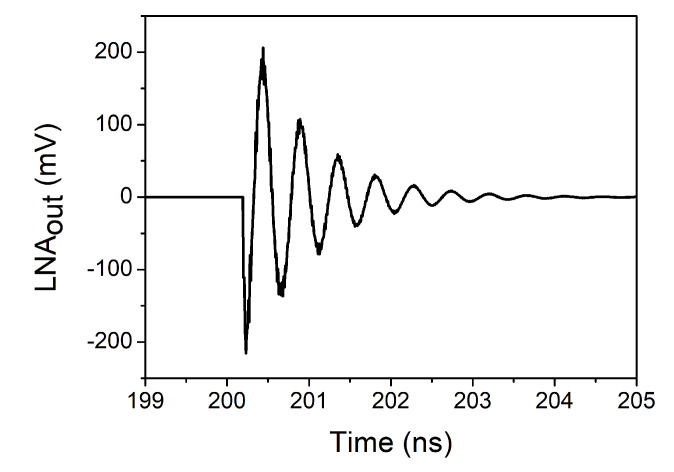
Effect of the pulse on the output of the LNA.

Figure 11 shows the observed signal at the output of the LNA. As it was stated in Section 3, it corresponds to an underdamped response. This can be proven by analysing the values of *α* and *ω*_0_. In this case, the values of L and C are 2.58 nH and 1.4 pF, respectively, while the quality factor (Q) of the tank is approximately 10.5, which results in a resistance of 464 Ω (see (4)). Taking this into account, and substituting in (2) and (3), it can be seen that in this case an underdamped response is obtained.

**Figure 12:**
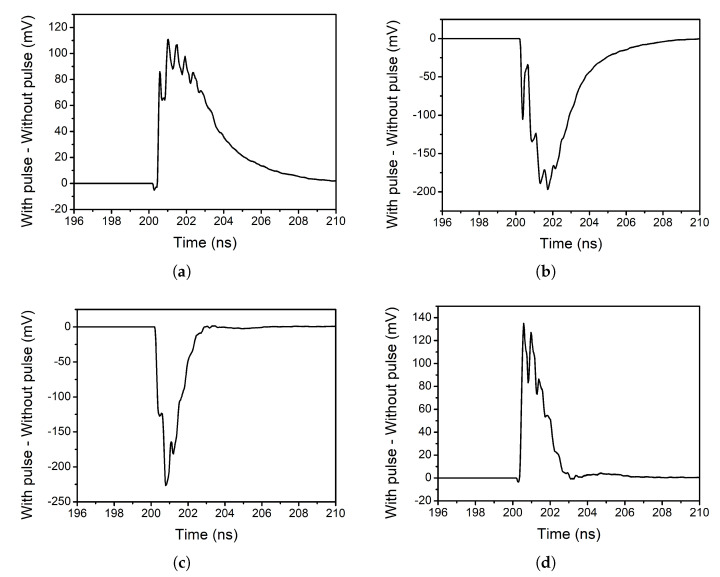
Effect of the pulse on the outputs of the mixer: (**a**) positive I branch, (**b**) negative I branch, (**c**) positive Q branch and (**d**) negative Q branch.

In order to study the effect of the mixer on the pulse, the signal at the output of the TIA is observed, once the current signal is converted to voltage. Therefore, in this study the outputs of the TIAs are considered to be the outputs of the mixer. Figure 12 shows the difference between the case of a strike occurring and the case when there is no strike, for each of the four outputs.

As it can be seen, the shape of the pulses that appear at the outputs of the mixer resembles the shape of the input pulse. However, the obtained pulses do not have the exact same shape as their original counterpart. This can be explained by the fact that the input pulses are very narrow (τ≈ 0.1 ns). Therefore, the condition presented in the previous section (5) is not met as *f_LO_* = 2.3975 GHz. Even though the pulse seen at the outputs of the mixer does not have the same shape as the input pulse, the obtained pulse has a width and amplitude that could be harmful for other circuits of the receiver chain.

The next element in the receiver chain is the complex filter. The designed filter is a Butterworth third-order gm-C complex filter. It was stated in the previous section that a filter effectively reduces the effect of pulses such as those implemented in this study. This is explained by the loss of spectral information during the filtering process as part of the pulse signal is suppressed.

**Figure 13:**
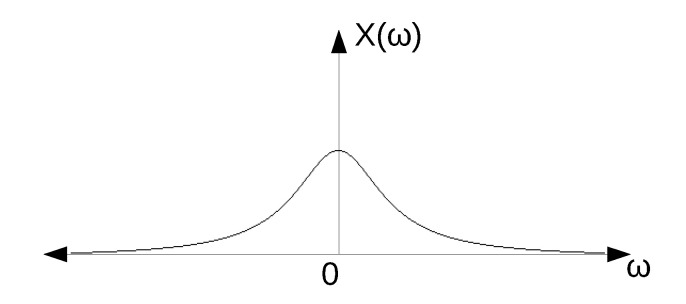
Double exponential pulse in the frequency domain.

Additionally, as it was previously mentioned, the bandwidth of a channel for low-power consumption standards is usually in the order of a few MHz. Specifically, for the IEEE 802.15.4 standard the channel bandwidth is 3 MHz. Taking into account that the frequency response of a double exponential pulse follows the shape sketched in Figure 13 [35], it can be stated that the complex filter is filtering most of the information of the double exponential pulse.

**Figure 14:**
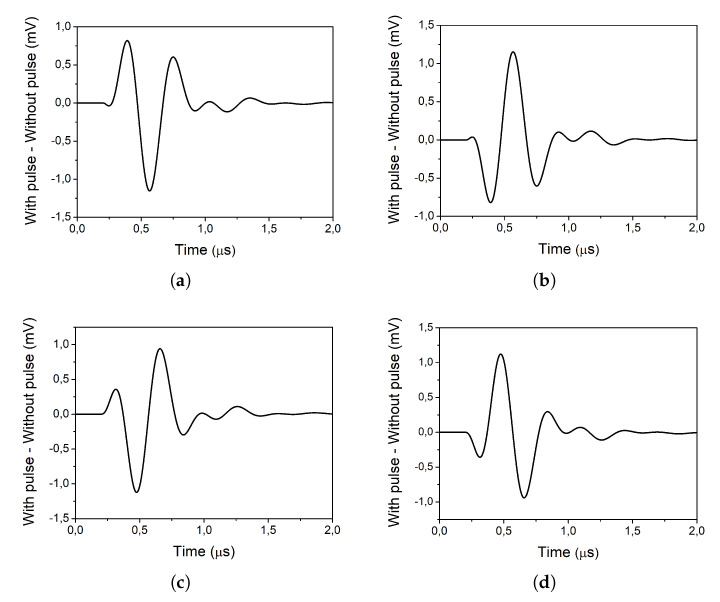
Effect of the pulse on the outputs of the complex filter (**a**) positive I branch (**b**) negative I branch (**c**) positive Q branch (**d**) negative Q branch.

This can be proven by observing the signal at the outputs of the complex filter. Figure 14 shows the difference between the case of a strike occurring and the case when there is no strike, for each of the four outputs. It can be seen that the pulse has been considerably minimised. The maximum voltage peak has been reduced from the order of hundreds of mV to approximately 1 mV.

**Figure 15:**
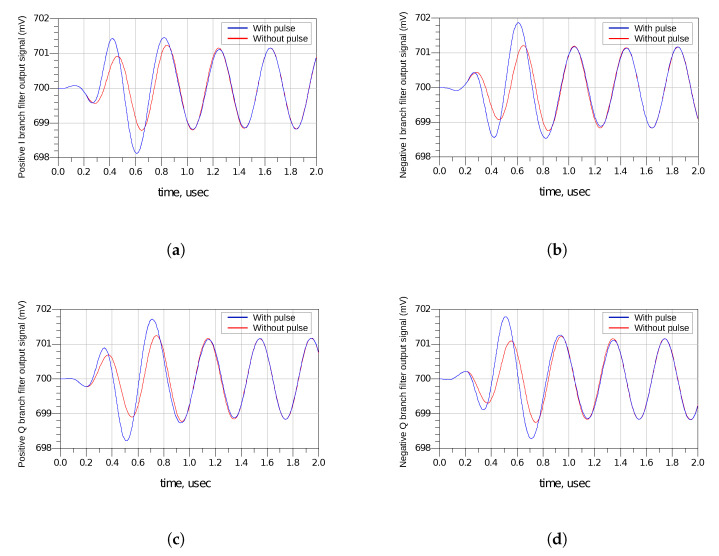
Output signals of the complex filter: (**a**) positive I branch, (**b**) negative I branch, (**c**) positive Q branch and (**d**) negative Q branch.

However, this minimised pulse can still cause harmful effects on the receiver, as it can be seen in Figure 15, which shows the voltage signal on the four outputs of the complex filter when there is a strike (blue) and when there is no strike (red) at the LNA.

It can be seen that there is a slight shift both in amplitude and phase at the output signals of the filter. This shift could result in a bit change in the digital circuits that follow the receiver front-end designed in this work, resulting in a slight increase in the bit error rate (BER) of the system. However, the severity of the SET is only known once it propagates until the end of the signal processing chain. The results obtained in the analysis performed in this paper allow the assessment of SETs in a whole RF system once the digital circuitry is included.

## 7. Conclusions

In this paper, the effect of single-event transients on an RF direct-conversion receiver has been analysed. In order to do so, it has been studied how current pulses generated in the low-noise amplifier propagate down the receiver chain. The effect of the different circuits that form the receiver on this kind of pulses has been studied prior to the analysis of the complete receiver.

First, the effect of SETs in low-noise amplifiers was analysed. Then, the propagation of the LNA-generated pulses through mixers was studied. To do so, double-balanced mixers were analysed under the effect of these pulses. Additionally, the effect of the duty cycle in the propagation of said pulses has been evaluated. Simulation results show that in all cases a pulse introduced in the input of the circuit will propagate to the output under certain conditions discussed in this paper.

The effect of filters on the analysed pulses has also been studied. The obtained results show that most part of the spectral information of the pulses could be lost in the filtering process, thus minimising the effect of SETs. In the case of low-IF receivers designed for low-power consumption standards, the bandwidth of a channel is usually in the order of a few MHz. Therefore, this spectral information loss will be further increased in the case of pulses with a width of approximately a few nanoseconds. Specifically, the filter implemented in the designed receiver effectively reduces the pulse as the maximum voltage peak is minimised from the order of hundreds of mV to approximately 1 mV.

Finally, the analysis of a low-IF down-conversion RF receiver was performed. The proposed procedure consists in analysing the voltage signal at different nodes of the receiver chain, checking for possible amplitude and phase variations in the signal. In the case of the analysed RF receiver, an amplitude and phase shift was observed under the presence of SETs.

## Abbreviations

The following abbreviations are used in this manuscript.

ADS	Advanced Design System
BER	Bit Error Rate
BPF	Band-Pass Filter
CMOS	Complementary Metal Oxide Semiconductor
LNA	Low-Noise Amplifier
LO	Local Oscillator
LPF	Low-Pass Filter
Q	Quality factor
RF	Radiofrequency
RHBD	Radiation Hardening By Design
SETs	Single Event Transients
SOI	Silicon-On-Insulator
TCAD	Technology Computer Aided Design
TIA	Transimpedance Amplifier
WSNs	Wireless Sensor Networks
